# Analysis of Apoptosis-Related Genes Reveals that Apoptosis Functions in Conidiation and Pathogenesis of *Fusarium pseudograminearum*

**DOI:** 10.1128/mSphere.01140-20

**Published:** 2021-01-06

**Authors:** Linlin Chen, Yuming Ma, Mengya Peng, Wenbo Chen, Huiqing Xia, Jingya Zhao, Yake Zhang, Zhuo Fan, Xiaoping Xing, Honglian Li

**Affiliations:** a State Key Laboratory for Rice Biology, Institute of Biotechnology, Zhejiang University, Hangzhou, China; b College of Plant Protection, Henan Agricultural University, Zhengzhou, China; c National Key Laboratory of Wheat and Maize Crop Science, Zhengzhou, China; University of Georgia

**Keywords:** *Fusarium pseudograminearum*, apoptosis, apoptosis-related genes, conidiation, pathogenesis

## Abstract

Apoptosis, a type of programmed cell death, plays crucial roles in various physiological processes, from development to adaptive responses. Key features of apoptosis have been verified in various fungal microbes but not yet in *Fusarium* species. Here, we identified 19 apoptosis-related genes in *Fusarium pseudograminearum* using a genome-wide survey. Expression profile analysis revealed that several apoptosis-related genes were significantly increased during conidiation and infection stages. Among these is FpBIR1, with two BIR (baculovirus inhibitor-of-apoptosis protein repeat) domains at the N-terminal end of the protein, a homolog of Saccharomyces cerevisiae BIR1, which is a unique apoptosis inhibitor. FpNUC1 is the ortholog of S. cerevisiae NUC1, which triggers AIF1- or YCA1-independent apoptosis. The functions of these two proteins were assessed by creating Δ*fpbir1* and Δ*fpnuc1* mutants via targeted gene deletion. The Δ*fpbir1* mutant had more cells with nuclear fragmentation and exhibited reduced conidiation, conidial formation, and infectivity. Correspondingly, the Δ*fpnuc1* mutant contained multiple nuclei, produced thicker and more branched hyphae, was reduced in conidiation, and exhibited faster conidial formation and higher infection rates. Taken together, our results indicate that the apoptosis-related genes *FpBIR1* and *FpNUC1* function in conidiation, conidial germination, and infection by *F. pseudograminearum*.

**IMPORTANCE** The plant-pathogenic fungus *F. pseudograminearum* is the causal agent of *Fusarium* crown rot (FCR) in wheat and barley, resulting in substantial yield losses worldwide. Particularly, in the Huanghuai wheat-growing region of China, *F. pseudograminearum* was reported as the dominant *Fusarium* species in FCR infections. Apoptosis is an evolutionarily conserved mechanism in eukaryotes, playing crucial roles in development and cell responses to biotic and abiotic stresses. However, few reports on apoptosis in plant fungal pathogens have been published. In this study, we identified 19 conserved apoptosis-related genes in *F. pseudograminearum*, several of which were significantly increased during conidiation and infection stages. Potential apoptosis functions were assessed by deletion of the putative apoptosis inhibitor gene *FpBIR1* and apoptosis trigger gene *FpNUC1* in *F. pseudograminearum*. The *FpBIR1* deletion mutant exhibited defects in conidial germination and pathogenicity, whereas the *FpNUC1* deletion mutant experienced faster conidial formation and higher infection rates. Apoptosis appears to negatively regulate the conidial germination and pathogenicity of *F. pseudograminearum*. To our knowledge, this study is the first report of apoptosis contributing to infection-related morphogenesis and pathogenesis in *F. pseudograminearum*.

## Abstract (precis)

The plant-pathogenic fungus *F. pseudograminearum* is the causal agent of *Fusarium* crown rot (FCR) in wheat and barley, resulting in substantial yield losses worldwide. Particularly, in the Huanghuai wheat-growing region of China, *F. pseudograminearum* was reported as the dominant *Fusarium* species in FCR infections.

## INTRODUCTION

The fungal species Fusarium pseudograminearum was initially differentiated from Fusarium graminearum via host tissue preferences and, based on molecular data, has been formally recognized as a separate species (1, 2). Additionally, F. pseudograminearum is heterothallic, while F. graminearum has a homothallic mating system. *F*. *pseudograminearum* has emerged as a primary causal agent of *Fusarium* crown rot (FCR) and *Fusarium* head blight (FHB) diseases in wheat and barley (3, 4). Infection of cereal crops with *F*. *pseudograminearum* can result in reductions in grain yield, with crop losses due to *Fusarium* crown rot reaching 9 to 89%, as well as grain quality due to mycotoxin contamination (5, 6). In China, *F*. *pseudograminearum*-derived crown rot was first detected in 2011, and an ∼70% incidence of diseased wheat exhibiting symptoms of crown rot was found in one surveyed field (7, 8). Genetic diversity within populations of *F*. *pseudograminearum* isolated from many parts of the world has been widely investigated (8–10). However, very few fungal pathogenicity factors have been identified in *F*. *pseudograminearum*; thus, reliable disease control strategies are lacking due to incomplete characterization of the molecular mechanisms contributing to pathogenesis by this organism.

Apoptotic cell death plays crucial roles in a multitude of physiological processes in eukaryotic organisms, including adaptive responses of cells to biotic and abiotic stresses (11, 12). In contrast to necrotic cell death, apoptosis refers to a constellation of characteristic changes, including cell shrinkage, membrane blebbing, chromatin condensation, and DNA fragmentation/degradation (13, 14). Apoptosis is a highly regulated process that cannot be stopped once it has begun. In Caenorhabditis elegans, a model for apoptosis, four genes comprise its core apoptosis pathway. The proteolytic caspase CED-3 is activated by the CED-4 protein, and the antiapoptotic B-cell lymphoma 2 (BCL-2) protein CED-9 functions upstream of CED-4 to prevent the activation of the CED-3 caspase. Upstream of CED-9 is a proapoptotic BH3-only protein, EGL-1 (15). The core apoptosis pathway in C. elegans has one or more homologous genes in mammals and human. Once extracellular or intracellular stimulus-triggered apoptosis is detected, the initiator caspases (caspases 8 and 9) are activated from inactive procaspases and go on to activate the executioner caspases (caspases 3, 6, and 7) (16, 17).

Recently, several apoptosis regulators have been identified and characterized in yeast. Similar to the mammalian apoptosis-inducing factor (AIF), in response to apoptosis stimuli such as aging or exposure to acetic acid or hydrogen peroxide (H_2_O_2_), yeast AIF1 translocates from the mitochondria to the nucleus, leading to nuclear condensation and DNA fragmentation (18, 19). However, key proteins involved in the progression of apoptosis are very different between humans and fungi. Homologs of human caspases are apparently absent in fungi; however, yeast bears the metacaspase YCA1 to respond to apoptotic stimuli (20, 21).

Inhibitor-of-apoptosis proteins (IAPs), characterized by the presence of one to three copies of the baculovirus IAP repeat (BIR) domain, were chiefly known for their ability to inhibit apoptosis by blocking caspase activation or activity (22–24). In yeast, BIR1 is the only known IAP. *BIR1* disruption results in higher cell death rates, and *BIR1* overexpression delays the onset of cell death during chronological aging (25, 26). BIR1 homologs have been identified in many filamentous fungi (27–29). In Botrytis cinerea, *BcBIR1* overexpression strains exhibited reduced programmed cell death (PCD) markers and produced enhanced disease symptoms in Phaseolus vulgaris and Arabidopsis thaliana, while Δ*bcbir1* mutant strains showed enhanced PCD and restricted lesions. As a defense, A. thaliana produces camalexin to induce B. cinerea PCD, demonstrated by *BcBIR1* overexpression and Δ*bcbir1* mutant strains showing reduced or enhanced sensitivity to camalexin, respectively, along with reduced or enhanced PCD (28). In Magnaporthe oryzae, MoBIR1 had antiapoptotic activity and was involved in reactive oxygen species (ROS) generation and pathogenicity (29).

As a hallmark of the apoptotic process, the caspase-activated DNase CAD (also called DNA fragmentation factor 40 [DFF40]) is responsible for DNA fragmentation in the core apoptosis pathway of mammals (30). In addition, endonuclease G (Endo G) as a mitochondrial nuclease has also been identified in cells undergoing an apoptotic process. Upon different stimuli, Endo G is released from mitochondria and translocated to the nucleus, where it cleaves chromatin DNA into nucleosomal fragments independently of caspases (31, 32). NUC1 is the yeast homolog of mammalian Endo G, and it also digests both DNA and RNA and efficiently triggers AIF1- or metacaspase YCA1-independent apoptotic cell death (33, 34). Upon apoptosis induction, NUC1 translocates to the nucleus, where it interacts with karyopherin Kap123p and histone H2B, causing DNA fragmentation (33, 35). The deletion of *NUC1* reduces cell death via apoptosis but increases mitochondrial respiration, thereby increasing necrotic cell death (33).

In the present work, we identified 19 apoptosis-related genes in *F*. *pseudograminearum*; several of these were significantly induced during conidiation and infection. Furthermore, we analyzed the unique putative apoptosis inhibitor FpBIR1 and a putative apoptosis inducer, FpNUC1, in *F*. *pseudograminearum*. An *FpBIR1* deletion mutant showed reductions in conidial germination and pathogenicity, whereas *FpNUC1* deletion enhanced conidial germination and pathogenesis of *F*. *pseudograminearum*. Apoptosis appears to negatively regulate conidial germination and pathogenicity of *F*. *pseudograminearum*.

## RESULTS

### Identification of apoptosis-related proteins in *F*. *pseudograminearum*.

**FIG 1:**
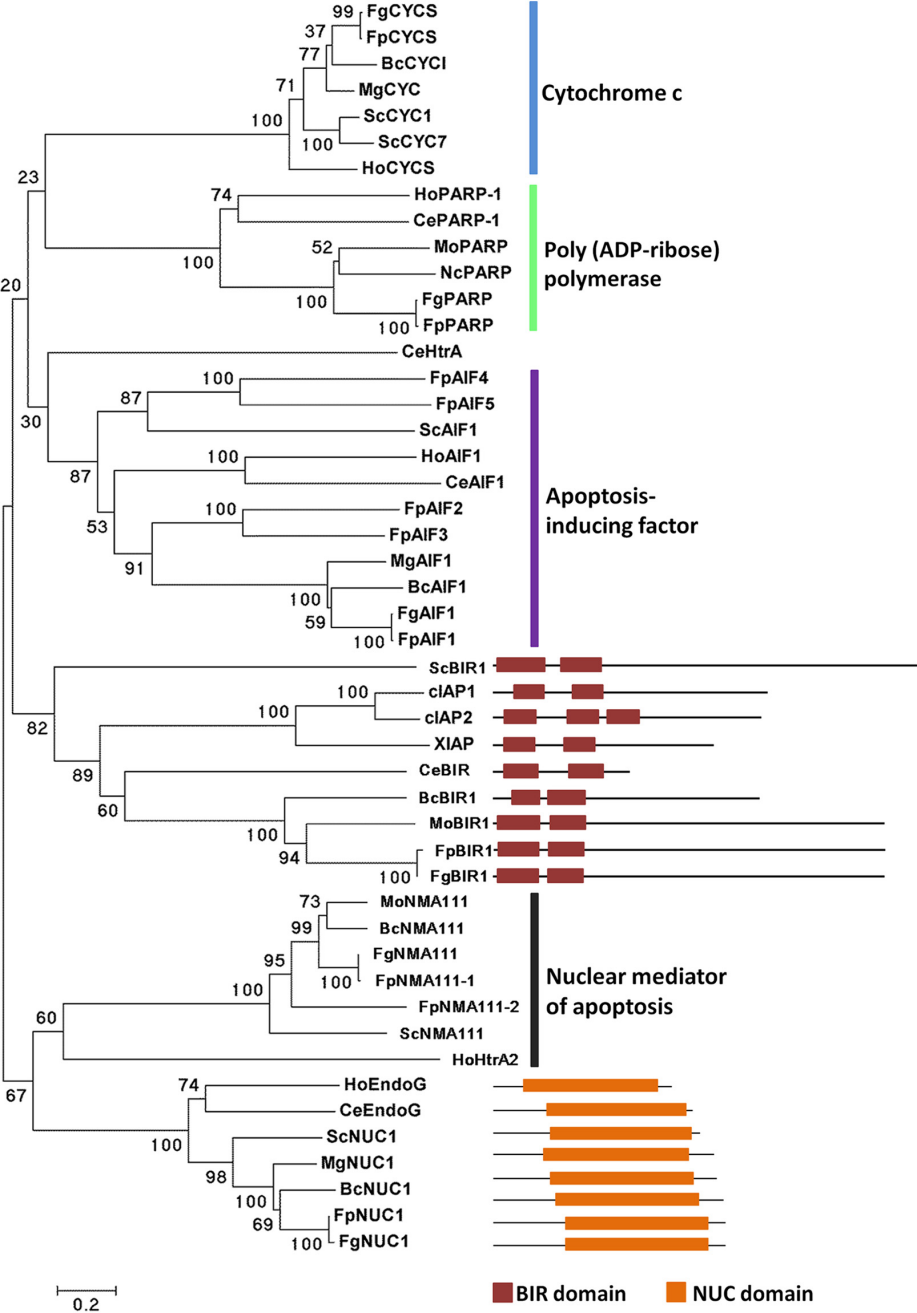
Phylogenetic tree analysis of apoptosis-related proteins. A neighbor-joining tree of apoptosis-related protein orthologs was constructed by using the MEGA 5 program, including H. sapiens (Ho), C. elegans (Ce), S. cerevisiae (Sc), *M. oryzae* (Mo), *B. cinerea* (Bc), Neurospora crassa (Nc), F. graminearum (Fg), and *F. pseudograminearum* (Fp). BIR and NUC domains were respectively predicted from IAP and Endo G orthologs.

Apoptosis-related proteins from Homo sapiens, C. elegans, Saccharomyces cerevisiae, and *B. cinerea* were used as the queries to search the candidate apoptosis-related protein homologs in *F*. *pseudograminearum*, and the deduced protein sequences were analyzed with the Pfam database to further confirm the putative homologs. A total of 19 putative apoptosis-related proteins were identified in *F*. *pseudograminearum* (Table 1; see also Data Set S1 in the supplemental material), and most of them seem to be conserved from fungi to human, including cytochrome *c*, the only known IAP in fungi; apoptosis-inducing factor; nuclear mediator of apoptosis homologs; poly(ADP-ribose) polymerase (PARP); nuclease Endo G; and TatD homologs (Fig. 1). Like other fungi, no caspase homologs were found, whereas four metacaspases were identified in *F*. *pseudograminearum*.

**TABLE 1:** Apoptosis-related proteins in *F*. *pseudograminearum*^*a*^

Gene name	Gene ID	Protein length (aa)	Conserved domain(s)	Mol wt (kDa)	pI
*FpCYCS*	FPSE_09824	106	Cytochrom_C	9.32	11.7
*FpBIR1*	FPSE_07259	874	BIR, BIR	5.44	95.4
*FpNUC1*	FPSE_01560	346	NUC	9.01	37.8
*FpMCA1*	FPSE_01129	418	Peptidase_C14	7.69	46.4
*FpMCA2*	FPSE_07591	402	Peptidase_C14	5.1	43.8
*FpMCA3*	FPSE_07894	649	Peptidase_C14	5.72	71.7
*FpMCA4*	FPSE_11435	644	Peptidase_C14	6.13	72.1
*FpAIF1*	FPSE_01973	540	Pyr_redox_2	6.20	57.3
*FpAIF2*	FPSE_00402	381	Pyr_redox_2	9.16	41.3
*FpAIF3*	FPSE_04328	372	Pyr_redox_2	6.52	39.8
*FpAIF4*	FPSE_03309	417	Pyr_redox_2	8.6	46.4
*FpAIF5*	FPSE_05514	394	Pyr_redox_2	8.94	43.3
*FpNMA111-1*	FPSE_11299	974	Trypsin_2, PDZ_1	5.29	108.4
*FpNMA111-2*	FPSE_10655	752	PDZ	6.74	83.1
*FpPARP*	FPSE_03056	330	BRCT, PARP	5.82	36.4
*FpTatD-1*	FPSE_08319	393	TatD_DNase	5.71	43.1
*FpTatD-2*	FPSE_06574	408	TatD_DNase	5.48	45.6
*FpTatD-3*	FPSE_07179	391	TatD_DNase	6.37	44.2
*FpTatD-4*	FPSE_05373	106	TatD_DNase	9.32	11.7

a aa, amino acids.

10.1128/mSphere.01140-20.3 DATA SET S1 Sequences of apoptosis-related proteins. Download Data Set S1, XLSX file, 0.03 MB. Copyright © 2021 Chen et al. 2021 Chen et al. This content is distributed under the terms of the Creative Commons Attribution 4.0 International license.

**FIG 2:**
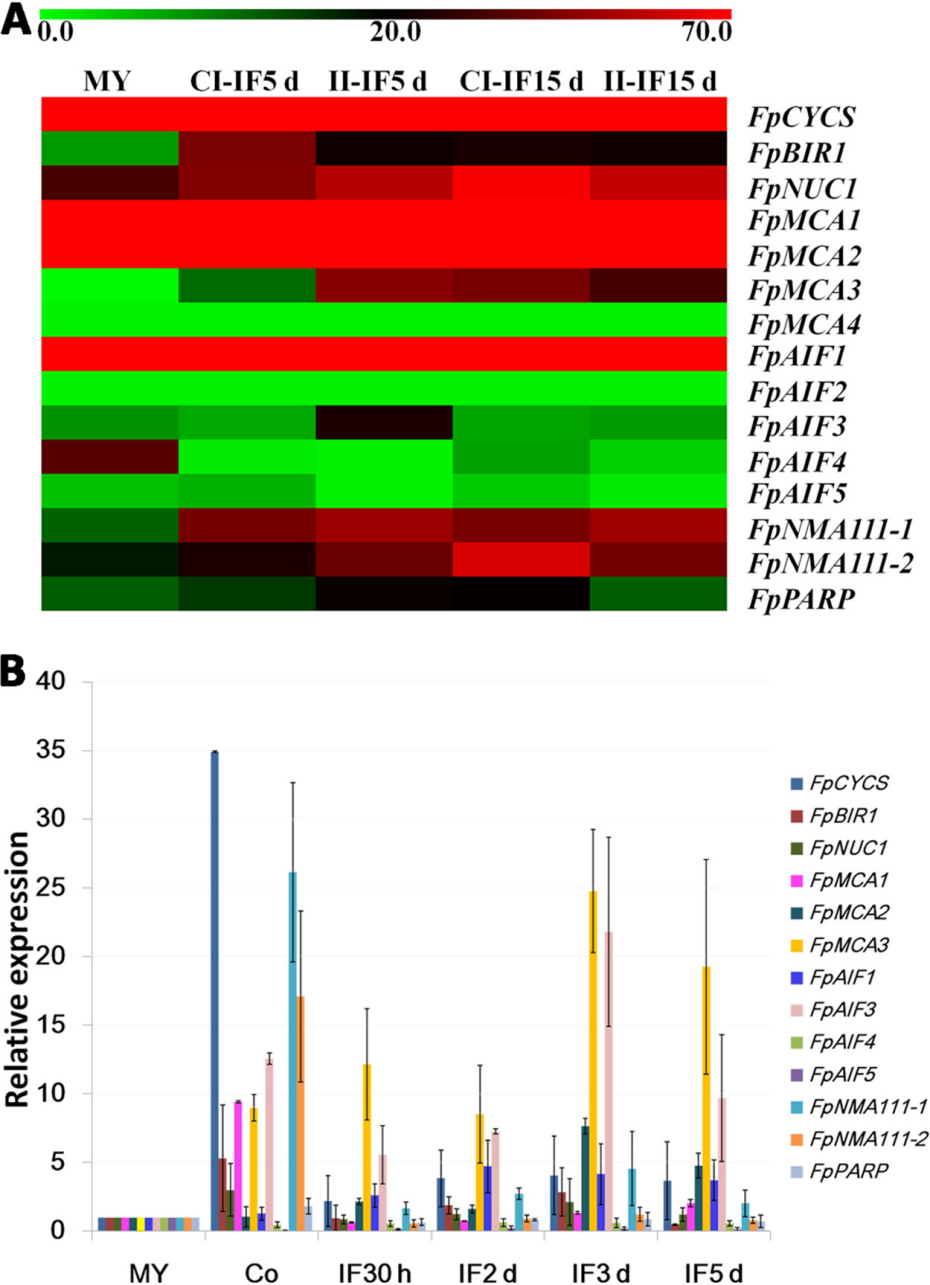
Expression profiles of apoptosis-related genes in *F*. *pseudograminearum*. (A) Transcriptome heat map. The color bar represents the expression values, ranging from green (0) to red (70). MY, mycelia. CI-IF5 d and CI-IF15 d indicate samples from 5 and 15 days after infection of susceptible wheat Guomai301; II-IF5 d and II-IF15 d indicate samples from 5 and 15 days after infection of resistant wheat Zhoumai24. (B) qRT-PCR analysis. Fold changes in transcript levels of the apoptosis-related genes were calculated relative to the level at mycelia (MY). Co, conidia. IF30 h, 2 d, 3 d, and 5 d indicate samples from susceptible wheat cultivar Aikang58 at 30 hpi, 2 dpi, 3 dpi, and 5 dpi, respectively. The *F. pseudograminearum TEF1* gene was used as a reference. Bars represent standard errors from three independent RNA isolations and qRT-PCR replicates.

Increasing evidence suggests that apoptosis is essential for the development and infectivity of many pathogenic organisms (36). To explore the potential role of apoptosis-related genes in the virulence of *F*. *pseudograminearum*, the expression patterns of 15 apoptosis-related genes in infection stages were obtained from a transcriptional database (37). Expression profiles of four *FpTatD* genes have been reported previously (38). Heat map analysis was performed based on the expression level of each gene (Fig. 2A; see also Table S2 in the supplemental material). Seven apoptosis-related genes, *FpBIR1*, *FpNUC1*, *FpMCA2*, *FpMCA3*, *FpAIF1*, *FpNMA111-1*, and *FpNMA111-2*, showed relatively higher expression levels during infection. Furthermore, the relative expression levels of apoptosis genes in *F*. *pseudograminearum* were validated by reverse transcription-quantitative PCR (qRT-PCR) (Fig. 2B). Comparable to the transcriptional data, *FpCYCS* showed the highest transcriptional level, while no amplification signal was detected in *FpMCA4* and *FpAIF2*. The levels of most apoptosis-related genes increased in conidia, and except for *FpMCA1*, *FpAIF4*, *FpAIF5*, *FpNMA111-2*, and *FpPARP*, transcriptional levels of other apoptosis-related genes were induced during infection stages. It was suggested that apoptosis might play roles in the conidiation and virulence of *F*. *pseudograminearum*.

### FpBIR1 and FpNUC1 are conserved in *F*. *pseudograminearum*.

According to protein domain predictions, FpBIR1 is the only theoretical inhibitor of apoptosis; FpNUC1 might trigger AIF- or metacaspase-independent apoptosis in *F*. *pseudograminearum*, and both *FpBIR1* and *FpNUC1* expression levels were induced in conidiation and infection stages. Thus, the potential effects of apoptosis in *F*. *pseudograminearum* were investigated by mutational analyses of *FpBIR1* and *FpNUC1*. In fungi, BIR1 of S. cerevisiae and BcBIR1 of *B*. *cinerea* belong to the type II (IAP-like) BIR-containing proteins, both of which have antiapoptotic activity mediated by the BIR domains at the N-terminal end of the protein. BIR1 is the only apoptosis inhibitor in fungi (2). Like these, *F*. *pseudograminearum* has a unique putative IAP that was assigned as FpBIR1. *FpBIR1* is 2,724 bp long and encodes 874 amino acids; it has two predicted introns, one 49 bp long, located between the 150th and 199th nucleotides, and one 50 bp long, located between the 603rd and 653rd nucleotides (Table 1). The *F*. *pseudograminearum* FpBIR1 protein contains two BIR domains at the N-terminal end of the protein (Fig. 1).

NUC1 (Endo G)-mediated apoptosis is independent of AIF1 or caspases/metacaspases in human and yeast (33, 39). The *FpNUC1* gene was identified as a 1,041-bp sequence lacking an intron and encoding a 346-amino-acid polypeptide (Table 1). A conserved NUC domain is predicted in the FpNUC1 protein (Fig. 1). Phylogenetic analysis revealed that NUC1 proteins were highly conserved in eukaryotes (Fig. 1), including the orthologs in human and yeast that have been reported as apoptosis inducers.

### Deletion of *FpBIR1* and *FpNUC1*.

**FIG 3:**
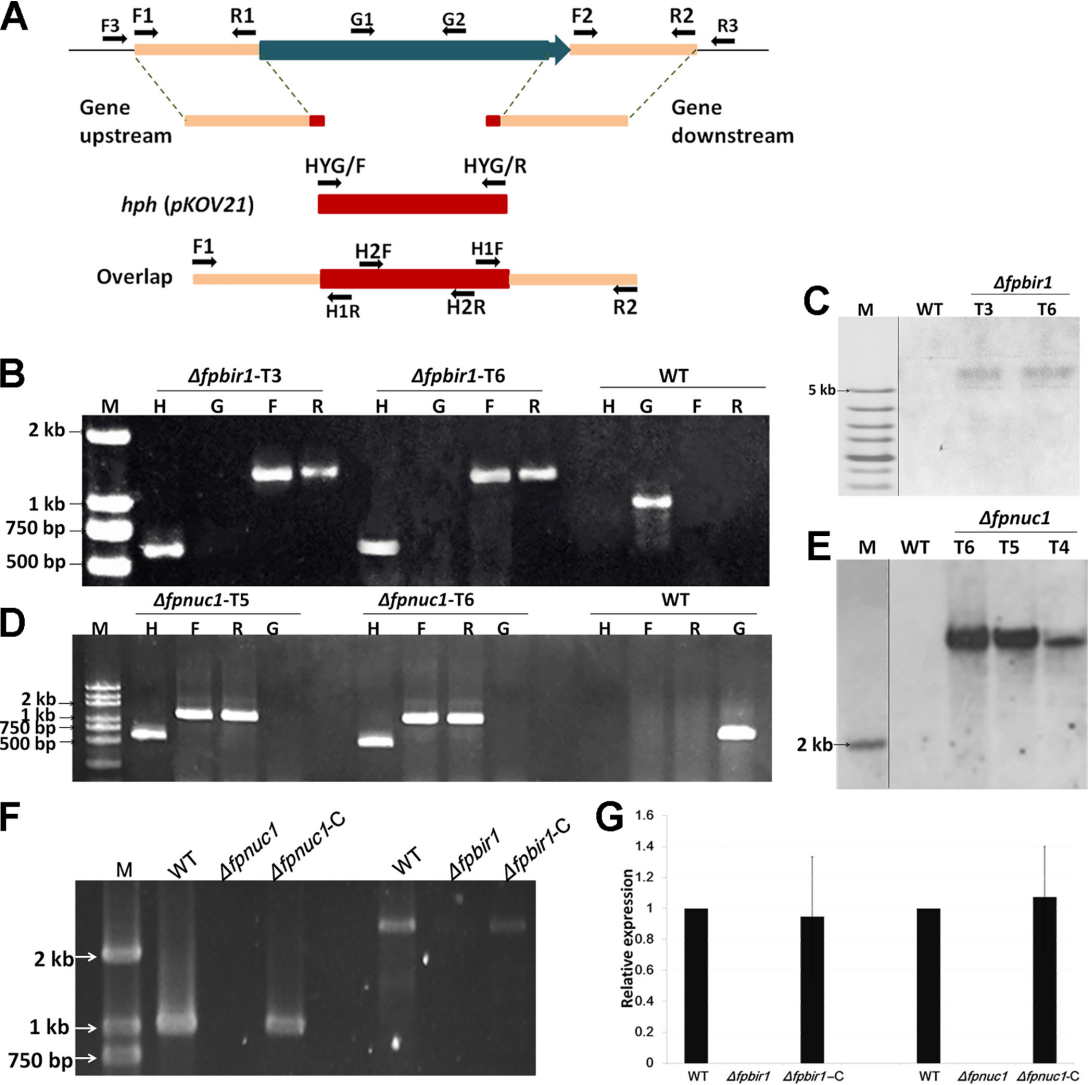
Construction of *FpBIR1* and *FpNUC1* deletion mutants. (A) Schematic representation of the genome regions of target genes and primer locations used for gene replacement using the split-marker strategy and mutant. (B and D) Verification of incorporation into genomic DNA by PCR using four primer pairs, which were used to analyze *hph* (H2F and H2R), upstream (F3 and H1R), downstream (H1F and R3), and target gene (G1 and G2) presence. Amplified fragments for *FpBIR1* replacement were 610 bp, 1,328 bp, 1,363 bp, and 638 bp. Amplified fragments for *FpNUC1* replacement were 610 bp, 1,292 bp, 1,348 bp, and 737 bp. WT, wild-type strain WZ 2-8A; M, molecular markers; H, *hph* gene; G, target gene; F, upstream; R, downstream. (C and E) Southern blot analysis using digoxigenin-labeled *hph* gene and XhoI-digested *FpBIR1* deletion mutant genomic DNA and BamHI-digested *FpNUC1* deletion mutant genomic DNA. (F) PCR products for the gene complementation constructs. A 2,625-bp fragment was amplified from the *FpBIR1* complementation strain, and a 1,041-bp fragment of *FpNUC1* was amplified from the *FpNUC1* complementation strain. (G) Expression levels of target genes assessed by qRT-PCR. Bars represent standard deviations from three independent RNA isolations and qRT-PCR replicates.

To obtain deletion mutants of *FpBIR1* and *FpNUC1*, target gene replacement constructs were generated by the split-marker approach and used to transform the wild-type (WT) strain WZ2-8A using the polyethylene glycol (PEG)-mediated protoplast stable transformation method (Fig. 3A). Transformants were first screened by PCR to identify mutants in which the deletion construct had been successfully integrated. Southern blot analysis was then used to confirm that the mutants contained a single copy of the gene disruption cassette. In total, two Δ*fpbir1* mutants (Fig. 3B and C) and two Δ*fpnuc1* mutants (Fig. 3D and E) were screened. To complement the deletion of *FpBIR1* and *FpNUC1*, the target gene with a green fluorescent protein gene (*GFP*) fusion construct driven by the gene promoter was generated. Complemented strains were identified via PCR and qRT-PCR (Fig. 3F and G). The FpBIR1 and FpNUC1 complemented strains were named Δ*fpbir1*-C and Δ*fpnuc1*-C, respectively.

### FpBIR1 and FpNUC1 regulate apoptotic activity.

**FIG 4:**
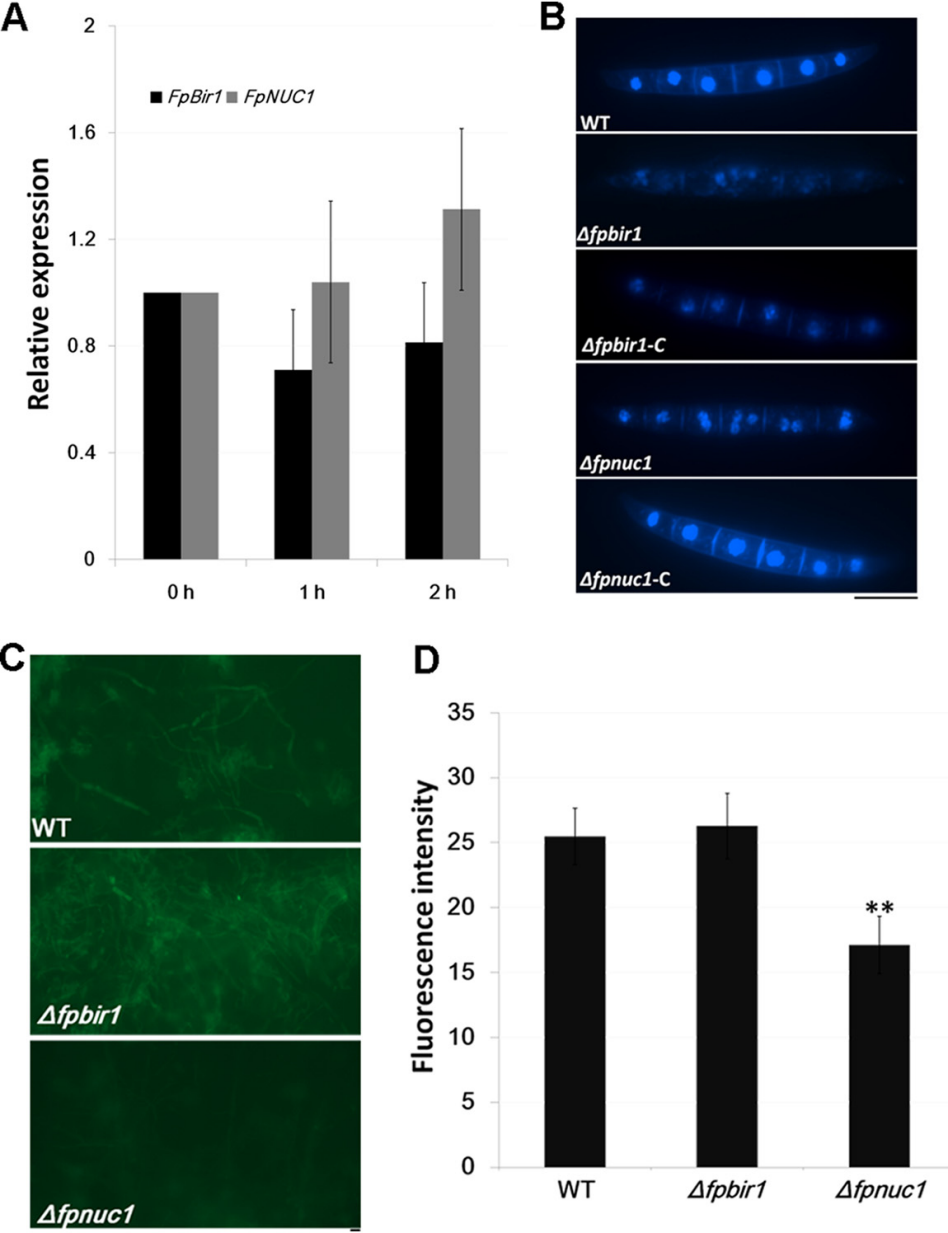
Apoptosis evaluation. (A) qRT-PCR analyses of *FpBIR1* and *FpNUC1* expression after *F*. *pseudograminearum* mycelium treatment with 5 mM H_2_O_2_ from 0 h to 2 h. Bars represent standard deviations from three independent RNA isolations and qRT-PCR replicates. (B) Fungal nuclear morphology of conidia was assessed by DAPI staining. Bar = 20 μm. (C) TUNEL staining of WT, Δ*fpbir1*, and Δ*fpnuc1* mycelia exposed to 5 mM H_2_O_2_ for 2 h. TUNEL-positive cells showed green fluorescence. Bar = 20 μm. (D) Green fluorescence intensity of TUNEL staining of WT, Δ*fpbir1*, and Δ*fpnuc1* mycelia. **, *P < *0.01 (by a *t* test).

H_2_O_2_, an important ROS, is a key mediator for eliciting PCD in mammalian and yeast cells (40, 41). To stimulate apoptosis, *F*. *pseudograminearum* mycelia were exposed to 5 mM H_2_O_2_ for 1 to 2 h. The qRT-PCR analysis showed that the expression of *FpBIR1* was decreased under H_2_O_2_ treatment, while *FpNUC1* expression increased (Fig. 4A). Nuclear morphology in fungal conidia was observed by 4′,6-diamidino-2-phenylindole (DAPI) staining. Compared with the WT and complemented strains, the Δ*fpbir1* mutants had more cells exhibiting nuclear fragmentation, which is a key feature of apoptosis, whereas Δ*fpnuc1* mutants contained multiple unfragmented nuclei (Fig. 4B). We then assessed DNA fragmentation in hyphae by terminal deoxynucleotidyltransferase-mediated dUTP-biotin nick end labeling (TUNEL) staining after treatment with H_2_O_2_. Compared with fluorescence in WT *F*. *pseudograminearum*, the Δ*fpbir1* mutant exhibited similarly strong fluorescence, whereas the Δ*fpnuc1* mutant showed only slight fluorescence, indicating a weaker degree of DNA fragmentation in this mutant (Fig. 4C and D).

### Deletion of *FpNUC1* affects mycelial growth and morphology.

**FIG 5:**
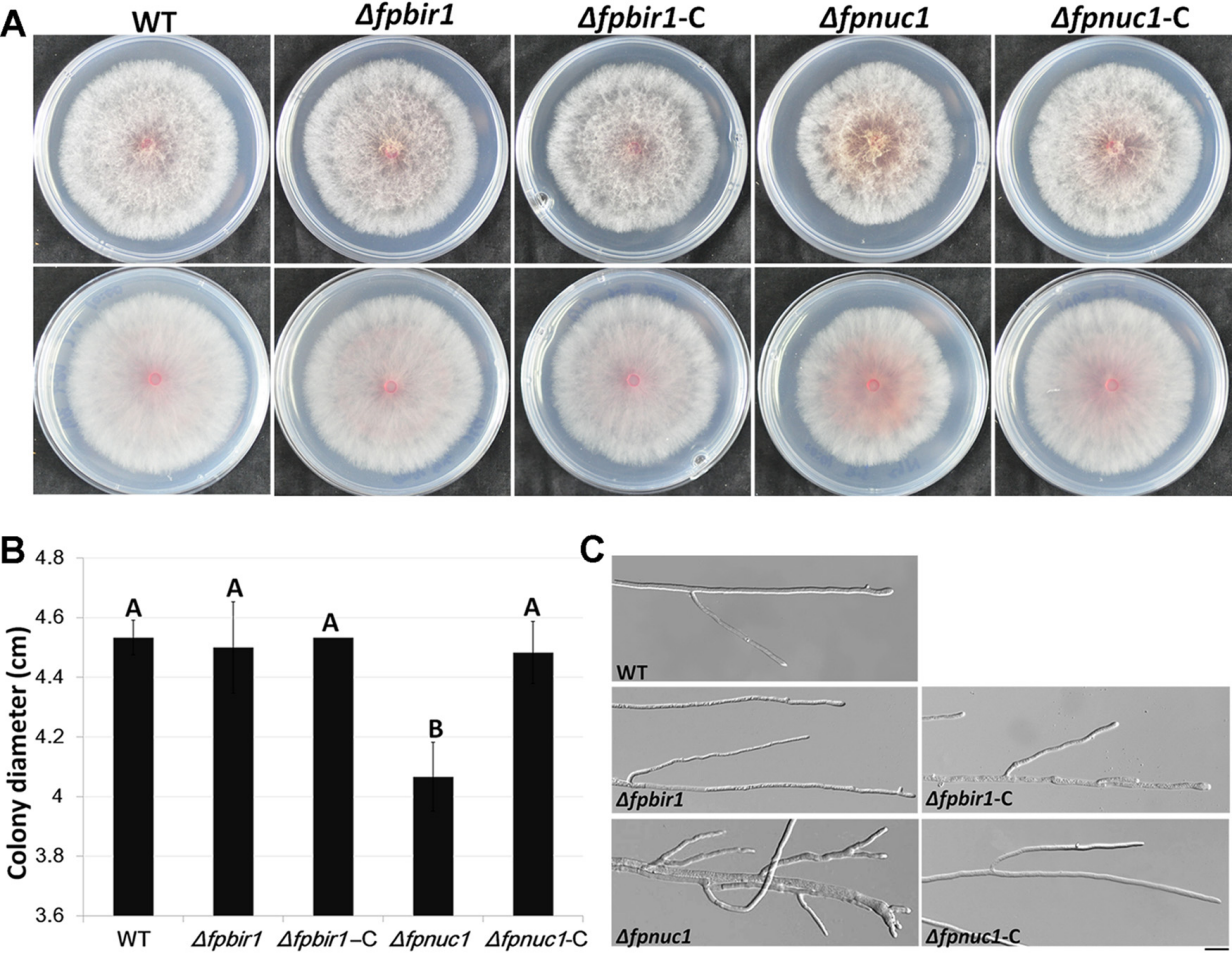
Colony morphology and hyphal growth. (A and B) Colonies of all five strains grown on PDA medium. Photographs and colony diameters were taken after 3 days. The data shown are representative of those from three separate experiments. A and B above bars indicate significant differences at a *P* value of <0.01 using Duncan’s multiple-range analysis. (C) Hyphae of all five strains cultured on PDA plates for 24 h. Bar = 20 μm.

To determine whether FpBIR1 and FpNUC1 affect the growth and development of *F*. *pseudograminearum*, growth rates of the wild type, the Δ*fpbir1* and Δ*fpnuc1* mutants, and the complemented strains in a potato dextrose agar (PDA) medium culture were analyzed. Growth rates of the Δ*fpbir1* mutants were similar to those of the WT and Δ*fpbir1*-C strains (Fig. 5A and B), but microscopic examination showed that the hyphae of the Δ*fpbir1* mutants were thinner and less branched (Fig. 5C). The average diameter of Δ*fpnuc1* colonies was smaller than those of other strains (Fig. 5A and B), and the colonies developed thicker, more branched mycelia (Fig. 5C). The opposite effects of FpBIR1 and FpNUC1 on mycelial morphology indicated that apoptosis might negatively regulate hyphal branching in *F*. *pseudograminearum*.

### Both FpBIR1 and FpNUC1 function in conidiation and conidial germination.

**FIG 6:**
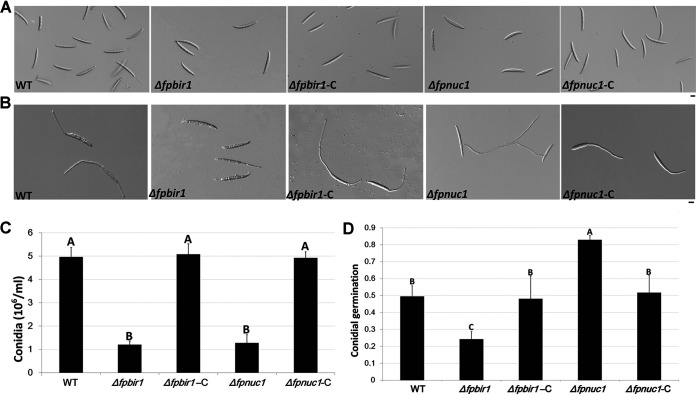
Conidial morphology and germination. (A) Conidial phenotypes. Micrographs were taken 3 days after mycelium induction in liquid CMC medium. Bar = 20 μm. (B) Conidial germination. Micrographs were performed 3 h after conidial induction in sterile distilled water. Bar = 20 μm. (C and D) Quantification of conidial production and germination of WT, Δ*fpbir1*, Δ*fpbir1*-C, Δ*fpnuc1*, and Δ*fpnuc1*-C strains. The data shown are representative of results from three separate experiments. The bars indicate standard errors. A and B above bars indicate significant differences at a *P* value of <0.01 using Duncan’s multiple-range analysis.

Conidiation of all strains was determined by culturing each strain in carboxymethylcellulose sodium salt (CMC) liquid medium, in the dark at 25°C, with shaking at 150 rpm for 4 days. Conidial production was quantified and microscopically observed (Fig. 6A and C). Relative to the WT strain, the numbers of conidia in Δ*fpbir1* and Δ*fpnuc1* mutants were decreased to ∼24% and ∼25%, respectively. No significant changes were detected among the WT, Δ*fpbir1*-C, and Δ*fpnuc1*-C strains. These results indicated that both FpBIR1 and FpNUC1 were important for conidiation.

Conidial germination was examined in sterile water (Fig. 6B and D). After incubation at 25°C for 3 h, approximately 50% of the WT conidia had germinated. Under the same conditions, only 24% of the Δ*fpbir1* mutant conidia had visible germ tubes. However, over 80% of the Δ*fpnuc1* mutant conidia produced germ tubes, which were longer than those of the wild-type conidia. The opposite effects of FpBIR1 and FpNUC1 indicated that apoptosis might negatively regulate the germination of *F*. *pseudograminearum* conidia.

### Both FpBIR1 and FpNUC1 are involved in virulence.

**FIG 7:**
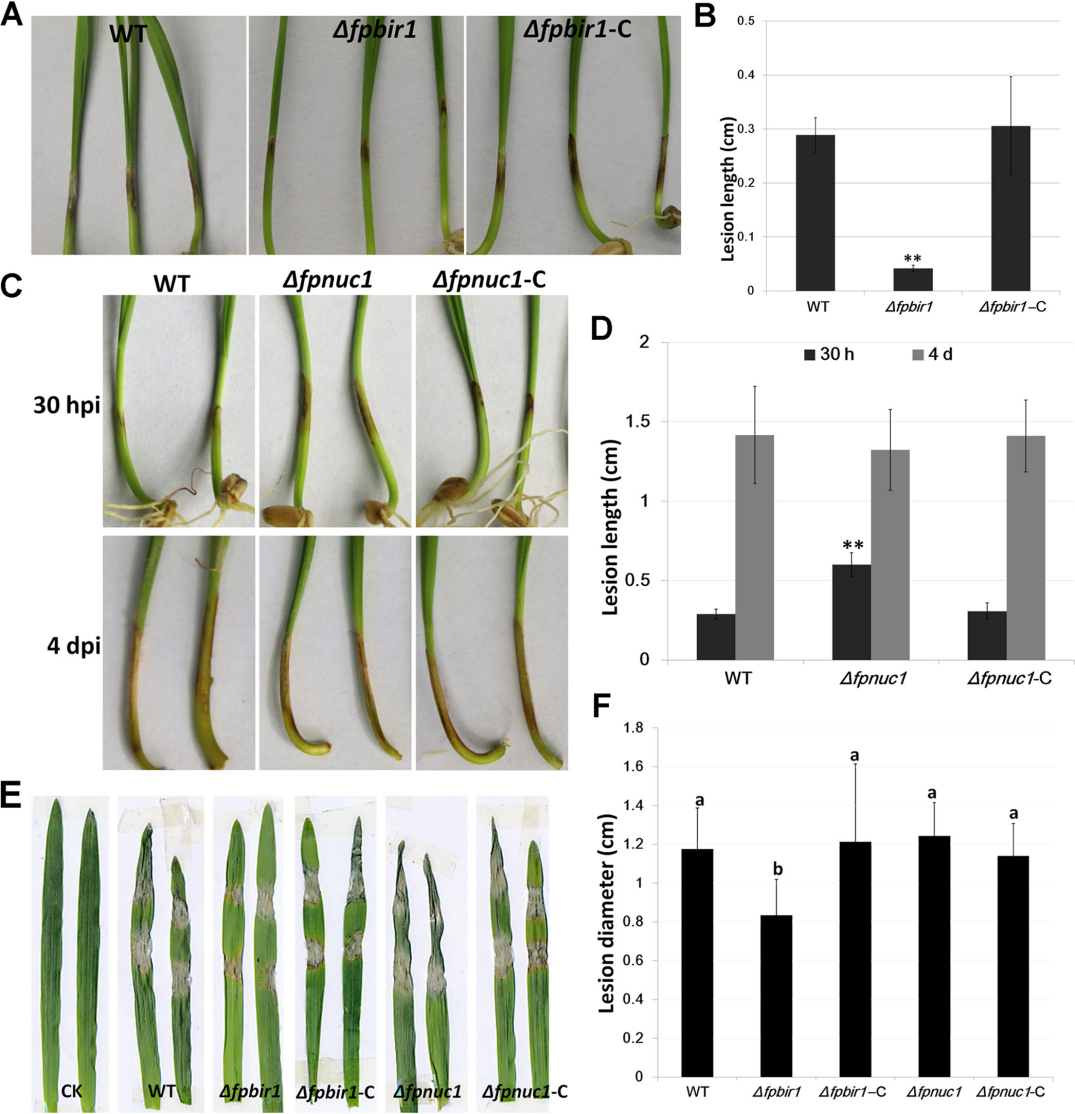
Infection assays. (A and B) Phenotypes and lesion lengths of wheat seedlings inoculated with mycelia of WT, Δ*fpbir1*, and Δ*fpbir1*-C strains. Photographs and lesion lengths were taken at 3 dpi. The data shown are representative of those from three separate experiments. Bars indicate standard errors. **, *P < *0.01 (by a *t* test). (C and D) Phenotypes and lesion lengths of wheat seedlings inoculated with mycelia of WT, Δ*fpnuc1*, and Δ*fpnuc1*-C strains. Photographs and lesion lengths were taken from 30 hpi to 3 dpi. The data shown are representative of results from three separate experiments. Bars indicate standard errors. **, *P < *0.01 (by a *t* test). (E and F) Phenotypes and lesion lengths of barley leaves inoculated with mycelia of WT, Δ*fpbir1*, Δ*fpbir1*-C, Δ*fpnuc1*, and Δ*fpnuc1*-C strains. Photographs and lesion lengths were taken at 3 dpi. CK indicates barley leaves inoculated with agar blocks. Bars indicate standard errors. a and b above bars indicate significant differences at a *P* value of <0.05 using Duncan’s multiple-range analysis.

Pathogenicity assays were conducted to determine the effects of the Δ*fpbir1* and Δ*fpnuc1* mutations on wheat coleoptiles and barley leaves infected with mycelia of these mutant strains. Upon infection with the Δ*fpbir1* mutant, lesion sizes were reduced by over 50% compared to the WT strain at 3 days postinoculation (dpi) (Fig. 7A and B). Wheat coleoptiles inoculated with the Δ*fpnuc1* mutant exhibited typical scab symptoms emerging at 20 h postinoculation (hpi). Compared with the WT and Δ*fpnuc1*-C strains, the Δ*fpnuc1* mutant induced virulence on wheat coleoptiles at 30 hpi. However, lesion expansion on wheat coleoptiles infected with the Δ*fpnuc1* mutant was slowed; there were no considerable differences among the WT, Δ*fpnuc1*, and Δ*fpnuc1*-C strains in wheat coleoptile infection at 4 dpi (Fig. 7C and D). The pathogenicity results for the Δ*fpnuc1* mutant were consistent with those for conidial germination.

A barley leaf inoculation assay was performed to validate these results (Fig. 7E and F). The Δ*fpbir1* mutants were less virulent than the WT and Δ*fpbir1*-C strains, whereas the Δ*fpnuc1* mutant exhibited normal infectivity compared with the WT and Δ*fpunc1*-C strains at 4 dpi. The opposite effects of FpBIR1 and FpNUC1 indicated that apoptosis might be a negative regulator of virulence in *F*. *pseudograminearum*.

## DISCUSSION

Programmed cell death (PCD) processes, including apoptosis, play prominent roles in plant-microbe interactions and may benefit either the host or the pathogen depending on the balance attained (42). Both plants and their pathogens attempt to manipulate each other using a complex network to maximize their respective survival probabilities. PCD in plants has been extensively studied, which is crucial in terms of resistance to pathogens (43). Pathogen resistance may also be influenced by pathogen viability in response to compounds produced by the plant host. For example, massive PCD of the necrotrophic fungus *B*. *cinerea* is induced by plant defense responses during the early phase of plant infection. In order to establish infection, antiapoptotic machinery is essential for protection of the fungus against host plant defenses during this stage (28). In Phytophthora sojae, apoptosis is inhibited in both early and late infection stages, likely playing different adaptive roles in implementing a successful infection cycle (44). Recent studies have shown that mammalian fungal pathogens also undergo apoptosis: Aspergillus fumigatus apoptosis-like cell death was triggered by lung neutrophils, where the pathogen opposes this process to cause disease in the murine lung (27). However, the role of PCD in *Fusarium* pathogens remains poorly understood, and the nature of regulation of apoptotic cell death is unknown. In the present work, we try to reveal a functional role of apoptosis in the development and pathogenesis of *F. pseudograminearum* through computational and mutational analyses of apoptosis-related genes.

In humans, apoptosis is a highly complex process that involves a cascade of molecular events controlled by different genes and molecular mechanisms (45). Extrinsic and intrinsic triggers activate caspases such as caspases 3, 6, and 7. Downstream caspases activate cytoplasmic endonucleases (such as caspase-activated DNase) leading to morphological changes characteristic of apoptosis, such as DNA fragmentation, the degradation of cytoskeletal and nuclear proteins, and the formation of apoptotic bodies. Cell death can be driven by a genetically programmed signaling pathway known as PCD (46–48). The understanding of apoptosis in plants and yeast has advanced in recent years, but the underlying regulatory mechanisms remain enigmatic. Animal apoptotic regulators, including caspases and BCL-2 family members, have not been identified in plants and yeast (13, 49). Similar to yeast, *F*. *pseudograminearum* lacks caspase and BCL genes, but several important apoptotic regulators found in yeast, such as AIF, Endo G, metacaspase, NMA111, and others, have also been identified in *F*. *pseudograminearum*, demonstrating that the basal apoptotic machinery is present in this organism. According to transcriptome and qRT-PCR analyses, the relative expression levels of several of these apoptosis-related genes were significantly increased during conidiation and infection stages, implying a putative role for apoptosis in the conidiation and virulence of this pathogen.

In fungi, BIR1 protects against apoptosis, and NUC1 induces AIF1- or YCA1-independent apoptosis; both genes are important for chromosome condensation, segregation, and spindle elongation during mitosis (14). In *F*. *pseudograminearum*, both *FpBIR1* and *FpNUC1* induced expression during conidiation and infection. Hydrogen peroxide stress reduced the expression of *FpBIR1* and induced the expression of *FpNUC1*. The results of DAPI staining and TUNEL analyses suggested that FpBIR1 has a conserved antiapoptotic function, while FpNUC1 positively regulates apoptosis.

By monitoring fungal physiology, apoptosis has been shown to play roles in fungal growth and development. Here, we found that both FpBIR1 and FpNUC1 were specifically related to conidial production and germination in *F*. *pseudograminearum*. The *FpBIR1* deletion mutant showed no change in the growth rate but experienced reduced conidiation and conidial germination rates. The *FpNUC1* deletion mutant exhibited thicker and more branched hyphae and increased conidial germination rates. However, the *FpNUC1* deletion mutant showed reduced mycelial growth rates, suggesting that *FpNUC1* might play roles in cell aging.

By monitoring fungal pathobiology, the antiapoptotic activity of the pathogen has been demonstrated to be important for host invasion by some fungi. AfBIR1 opposes apoptosis-like features in A. fumigatus, triggered by lung neutrophils, by inhibiting fungal caspase activation and DNA fragmentation in the murine lung (27). BcBir1 is a major regulator of PCD in *B*. *cinerea*, and proper regulation of host-induced PCD is essential for pathogenesis (28). Apoptosis induction in *PsTatD*-overexpressing transformants of P. sojae resulted in significant reductions in pathogenicity (44). Mutant strains lacking *FpBIR1* exhibited decreased virulence in wheat, while *FpNUC1* deletion mutants showed higher rates of infection. However, many questions remain to be answered in future research, such as what regulatory mechanisms exist for FpBIR1 and FpNUC1 in apoptosis and other physiological processes.

## MATERIALS AND METHODS

### Apoptosis-related protein identification in *F*. *pseudograminearum*.

To identify apoptosis-related proteins in *F*. *pseudograminearum*, the known apoptosis proteins were identified from GenBank, including 18 proteins of H. sapiens, 9 proteins of C. elegans, 7 proteins of S. cerevisiae, and 5 proteins of *B. cinerea*. Databases for *F*. *pseudograminearum* were obtained from the original source (50). Reciprocal BLASTP analysis was used with an *E* value cutoff of 1e−5. Homologs of M. oryzae and F. graminearum were subjected to a BLAST search and downloaded from the NCBI database. Significant hits were assessed for the presence of an obvious apoptosis-related functional domain using Pfam (51). The molecular weight and isoelectric point values of apoptosis-related proteins were calculated by using the ExPASy bioinformatics resource portal (www.expasy.org). The phylogenetic tree of apoptosis-related protein homologs was generated using MEGA 5 by the neighbor-joining method with 1,000 replicates for bootstrap analysis (52).

### Gene expression analysis.

For transcriptome analyses, mycelia were harvested by conidium cultivation in potato dextrose liquid medium at 25°C in darkness for 12 h. A pot culture experiment was used to harvest the infection samples. Sterile millet was inoculated with *F*. *pseudograminearum* mycelia, incubated at 25°C for 7 days, and then mixed with sterile soil (0.5% inoculation millet), in which wheat was subsequently grown. For negative controls, sterile millet was used. After 5 and 15 days, wheat roots from each pot were collected and washed thoroughly under running tap water and distilled water so that no soil particles remained. Samples of 6 μg of total RNA were extracted from each of the above-mentioned samples using the total RNA purification system (Invitrogen, Carlsbad, CA, USA). The total transcriptome was sequenced by the Gene Denovo Company (Guangzhou, China). Transcriptome raw data have been submitted and are publicly available in the NCBI database (accession number SUB5545839) (37).

### Quantitative RT-PCR.

To analyze apoptosis-related gene expression during infection, conidia of the wild type were collected in carboxymethylcellulose sodium salt (CMC) liquid, and wheat coleoptiles were inoculated with conidial suspensions of 1 × 10^7^ conidia/ml for 30 h, 2 days, 3 days, and 5 days. To test the expression levels of *FpBIR1* and *FpNUC1* under H_2_O_2_ treatment, mycelia of the indicated strains were cultured in yeast extract-peptone-dextrose (YEPD) medium with 5 mM H_2_O_2_ for 1 to 2 h at 25°C and shaken at 150 rpm. Total RNA was extracted from lyophilized mycelia using the EASYspin plant RNA kit (Aidlab, China) according to the manufacturer’s instructions. For RT-PCR, RNA was reverse transcribed into first-strand cDNA with a reverse transcription kit (TaKaRa, Dalian, China). qRT-PCR was performed with an Applied Biosystems 7000 real-time PCR system using SYBR green dye for fluorescence detection. The relative quantification transcript levels of the *FpBIR1* and *FpNUC1* genes were normalized to that of the *F. pseudograminearum TEF1*. All primers used in this study are listed in Table S1 in the supplemental material.

10.1128/mSphere.01140-20.1 TABLE S1 Primers used in the study. Download Table S1, DOCX file, 0.02 MB. Copyright © 2021 Chen et al. 2021 Chen et al. This content is distributed under the terms of the Creative Commons Attribution 4.0 International license.

10.1128/mSphere.01140-20.2 TABLE S2 Transcriptome data (fragments per kilobase per million [FPKM] values) of apoptosis-related genes in *F. pseudograminearum*. Download Table S2, DOCX file, 0.01 MB. Copyright © 2021 Chen et al. 2021 Chen et al. This content is distributed under the terms of the Creative Commons Attribution 4.0 International license.

### Fungal transformation and confirmation.

The split-marker approach was used to generate gene replacement constructs for the *FpBIR1* and *FpNUC1* genes. A schematic diagram of the primers used for generating deletion mutants and PCR amplification are shown in Fig. 3A, and the primers are listed in Table S1. The ∼1,000-bp upstream and downstream flanking genomic sequences of target genes were amplified with primer pair F1 and R1 and primer pair F2 and R2, respectively. The hygromycin phosphotransferase gene (*hph*) was amplified from plasmid pKOV21 by using primer pair HYG/F and HYG/R. Finally, the overlapping fragments containing the “target gene upstream-*hph* (pKOV21)-target gene downstream” cassette were obtained by overlap PCR amplification with primer pair F1 and R2 using mixed fragments of the upstream and downstream target genes and *hph* fragments as the templates. The resulting PCR products were purified and stored at −20°C for protoplast transformation. Polyethylene glycol (PEG)-mediated protoplast fungal transformation was performed as described previously (53).

Genomic DNA was isolated from mycelia (54) and screened for putative gene deletions by PCR using the primers H2F and H2R, F3 and H1R, H1F and R3, and G1 and G2. Southern blot analysis was performed with digoxigenin (DIG) high prime DNA labeling and detection starter kit I (Roche, Mannheim, Germany). The full-length target genes without stop codons together with their ∼1,500-bp promoter regions were amplified from the WT genome via PCR, ligated into the pKNTG vector, and introduced into the confirmed deletion strains by PEG-mediated transformation. The complementation strains were verified by PCR and qRT-PCR.

### TUNEL assays.

*F*. *pseudograminearum* mycelia were incubated in 5 mM H_2_O_2_, washed with phosphate-buffered saline (PBS) 2 h later, and assayed using one-step TUNEL apoptosis assay kits (catalog number C1086; Beyotime) according to the manufacturer’s protocol. Stained cells were imaged using a Carl Zeiss Axio imager M2 microscope registering fluorescence at 450 to 490 nm. Fluorescence intensity was assessed using ImageJ.

### Strains and growth conditions.

All the *F*. *pseudograminearum* strains used in this study were cultured on potato dextrose agar (PDA) plates at 25°C and stored in 30% glycerin at −70°C. To assess mycelial growth and colony characteristics, the wild-type strain WZ2-8A and mutants were cultured on PDA plates at 25°C. Mycelial morphology was observed at 24 h, and colony diameters were measured 3 days later; all experiments were performed at least three times, with over four replicates in each experiment.

### Conidiation and conidial germination assays.

To assess conidiation, three agar blocks (5 mm in diameter) carrying mycelia were introduced into 100 ml of liquid CMC medium. The suspension was shaken at 150 rpm for 4 days, conidia were filtered with one layer of miracloth, and the concentration of conidia was determined using a hemocytometer. The experiments were performed three times with 10 counting repetitions. Cell nuclei were stained with 1 μg/ml DAPI in the dark, observed at a magnification of ×600 under a fluorescence microscope (Nikon Eclipse Ti-S; Nikon, Tokyo, Japan) connected to a digital camera (Nikon Digital Sight DS-Ri2; Nikon, Tokyo, Japan), and analyzed using NIS-elements 4.5 imaging software (Nikon, Tokyo, Japan). For conidial germination assays, conidia were resuspended to 5 × 10^4^ conidia/ml in sterile water, and 50-μl droplets of the suspensions were placed on glass coverslips (Fisher Scientific) and incubated at 25°C for 3 h.

### Pathogenicity assays.

Virulence levels were determined on 7-day-old wheat cv. aikang58 seedings or 10-day-old malting barley leaves, both highly susceptible to *F*. *pseudograminearum*. Mycelial plugs of the indicated strains were placed onto intact wheat coleoptiles or barley leaves. Inoculated seedlings or leaves were kept in high humidity at 25°C in darkness. The mycelial plugs were removed after 24 h. Each assay was repeated three times. Disease severity was assessed.
