# Supplementary material for: Analysis of Apoptosis-Related Genes Reveals that Apoptosis Functions in Conidiation and Pathogenesis of Fusarium pseudograminearum
Source: mSphere. 2021 Jan 6;6(1):e01140-20. doi: 10.1128/mSphere.01140-20 (PMC7845595; doi:10.1128/mSphere.01140-20)
Supplement: TABLE S1 [file mSphere.01140-20_st001.docx]

**Table S1. Primers used in the study**

| Primer | Sequence (5΄→3΄) |
| --- | --- |
| FpBIR1-F1 | TTACCCCCATAAACCCGGGCTC |
| FpBIR1-R1 | CAATATCATCTTCTGTCGACTGGCTGTAGGTAGGTTGCGC |
| FpBIR1-F2 | TCCGAGGGCAAAGGAATAGGACATGACTTTCTGGAGAAC |
| FpBIR1-R2 | GTACAATACATCATCTACGATAC |
| HYG/F | GGCTTGGCTGGAGCTAGTGGAGGTCAA |
| HYG/R | GGAACCCGCGGTCGGCATCTACT |
| FpBIR1-F3 | CCGTTCGAAAATTCTTTACCTC |
| H1R | GCTGATCTGACCAGTTGC |
| H1F | GTCGATGCGACGCAATCGT |
| FpBIR1-R3 | ATCTTCGATAGCTTTGACGTAG |
| H2F | AACTCACCGCGACGTCTGTC |
| H2R | TTGTCCGTCAGGACATTGTT |
| FpBIR1-G1 | AGTGTTATGACAACAGCCTCG |
| FpBIR1-G2 | TCGTCCTCATCTGGCTCAATG |
| FpBIR1-cpF | GGGGTACCACGTTGGACGATTCACTGTGTAC |
| FpBIR1-cpR | CCATCGATTTCCACAACCAGTCCTTCCAG |
| FpBIR1-F | ATGTCTTTCGACGACATTACCGAC |
| FpBIR1-R | TCATTCCACAACCAGTCCTTCCAG |
| FpNUC1-F1 | GGGAGGAATGCGTTATTGACAAG |
| FpNUC1-R1 | CAATATCATCTTCTGTCGACGGTTGCTCTTGGAGGTGCCT |
| FpNUC1-F2 | ATAGAGTAGATGCCGACCGCGGGTTCATCAGATATGCTGTATAGTAG |
| FpNUC1-R2 | ATTGGAGAAGCAGCCGCAGAAGC |
| FpNUC1-F3 | CATCGAGTATCGAGATAAGGAG |
| FpNUC1-R3 | GATCCGACCACAGTACCTGCGCA |
| FpNUC1-G1 | AGGTGGTAAACGCGTCGAGGAG |
| FpNUC1-G2 | TTCCACCAGCACGACCGTCCT |
| FpNUC1-cpF | GGGGTACCTGAGAACCTCTATGAGTTGGAG |
| FpNUC1-cpR | CCATCGATCTTCTTCGGGAAAGCTTTTTGTC |
| FpNUC1-F | ATGTCCAAGACTACACTCGCTAC |
| FpNUC1-R | TTACTTCTTGGGGAAAGCTTTTTG |
| FpBIR1-RTF | TGATGCCGAGAACGAACCAC |
| FpBIR1-RTR | TTGCGTTTGGATGGTGACAG |
| FpNUC1-RTF | CGCACTTTGAGGATTTCTG |
| FpNUC1-RTR | TCTCATACTTGACATACCAC |
| FpMCA1-RTF | CAGATACTCAAACTGCAC |
| FpMCA1-RTR | CATATCCTCGCGCTTGTAAC |
| FpMCA2-RTF | CTATGGCTACAAGAGAGAG |
| FpMCA2-RTR | GGAAAAGAGCATCATCAGG |
| FpMCA3-RTF | AGTTCATACGTATCCTTGAAG |
| FpMCA3-RTR | GTATCGGTCACGTTTGGCCTT |
| FpMCA4-RTF | TCGAACACATAGTGCGATAC |
| FpMCA4-RTR | GAAGATGTCTGCGGTTCGAGT |
| FpCYCS-RTF | AAGACCCGATGTGCTCAGT |
| FpCYCS-RTR | TACCCTTCTGCTTGTTGG |
| FpAIF1-RTF | TCTGTCAAGGTCAATGACGAC |
| FpAIF1-RTR | AACTGGCTTGCCTTCACCTC |
| FpAIF2-RTF | AACGCTATTCCTGACCAAC |
| FpAIF2-RTR | AGGCCACATTGATACTGC |
| FpAIF3-RTF | TCGCACGAAGAGCTTGTA |
| FpAIF3-RTR | GAGATCAGAAGGACAGTG |
| FpAIF4-RTF | CTCGCAATACCGTCTTCT |
| FpAIF4-RTR | CTGTGTTGACGTGTGACT |
| FpAIF5-RTF | GACATATCAGCAGGGTATC |
| FpAIF5-RTR | TCGAGTGAACCAAGGTAAC |
| FpNMA111-1-RTF | CAATGCCAATTCCTTCTC |
| FpNMA111-1-RTR | CCTCGATCTTCTTGTCTG |
| FpNMA111-2-RTF | TCTCGTACCAGGTTGCACA |
| FpNMA111-2-RTR | GTTTCCAGGTTAGGTGTCT |
| FpRARP-RTF | GAAGACGTGAAGGAGGAA |
| FpRARP-RTR | TGGTTCAGTGAGGCATCA |
| TEF1-RTF | TCACCACTGAAGTCAAGTCC |
| TEF1-RTR | ACCAGCGACGTTACCACGTC |
