# Supplementary material for: Analysis of Apoptosis-Related Genes Reveals that Apoptosis Functions in Conidiation and Pathogenesis of Fusarium pseudograminearum
Source: mSphere. 2021 Jan 6;6(1):e01140-20. doi: 10.1128/mSphere.01140-20 (PMC7845595; doi:10.1128/mSphere.01140-20)
Supplement: TABLE S2 [file mSphere.01140-20_st002.docx]

| Gene Name | MY (replication 1) | MY (replication 2) | CI-IF 5d (replication 1) | CI-IF 5d (replication 2) | II-IF 5d (replication 1) | II-IF 5d (replication 2) | CI-IF 15d (replication 1) | CI-IF 15d (replication 2) | II-IF 15d (replication 1) | II-IF 15d (replication 2) |
| --- | --- | --- | --- | --- | --- | --- | --- | --- | --- | --- |
| *FpCYCS* | 3854.44 | 2661.82 | 1086.49 | 1931.23 | 1799.48 | 1311.07 | 498.23 | 1115.83 | 941.77 | 2055.32 |
| *FpBIR1* | 7.2 | 8.78 | 57.27 | 31.72 | 13.63 | 32.7 | 24.79 | 25.64 | 20.31 | 26.95 |
| *FpNUC1* | 30.57 | 37.6 | 55.9 | 34.92 | 62.08 | 50.06 | 55.59 | 82.56 | 78.46 | 38.32 |
| *FpMCA1* | 141.2 | 176.95 | 223.9 | 142.41 | 145.77 | 429.81 | 226.44 | 156.62 | 177.34 | 87.73 |
| *FpMCA2* | 288.82 | 354.33 | 259.7 | 152.9 | 398.3 | 0 | 393.27 | 401.97 | 270.42 | 341.27 |
| *FpMCA3* | 0.48 | 0.25 | 16.05 | 7.12 | 25.45 | 68.78 | 60.22 | 26.53 | 54.49 | 12.59 |
| *FpMCA4* | 0 | 0 | 0 | 0 | 0 | 0 | 0 | 0 | 0 | 0 |
| *FpAIF1* | 240.02 | 197.72 | 394.55 | 444.13 | 327.55 | 113.92 | 291.52 | 193.73 | 309.24 | 270.24 |
| *FpAIF2* | 0.43 | 1.55 | 0 | 0 | 0 | 0 | 0 | 0 | 0 | 0 |
| *FpAIF3* | 9.79 | 7.01 | 6.35 | 7.05 | 6.27 | 45.45 | 3.69 | 10.37 | 12.28 | 3.32 |
| *FpAIF4* | 36.8 | 37.51 | 0 | 3.04 | 0 | 0 | 1.06 | 13.46 | 2.12 | 5.01 |
| *FpAIF5* | 3.41 | 6.11 | 11.78 | 0 | 0 | 0 | 3.43 | 4.82 | 0 | 3.08 |
| *FpNMA111-1* | 10.22 | 14.18 | 38.97 | 47.48 | 59.94 | 41.7 | 71.89 | 16.18 | 79.58 | 23.43 |
| *FpNMA111-2* | 12.37 | 23.44 | 34.55 | 16.88 | 38.31 | 43.5 | 94.21 | 31.2 | 60.29 | 25.23 |
| *FpRARP* | 14.44 | 10.91 | 18.98 | 12.02 | 5.38 | 38.72 | 32.5 | 10.1 | 7.32 | 18.08 |

**Table S2.** Transcriptome data (FPKM values) of apoptosis related genes in *F. pseudograminearum*
